# When there is noise on Sherlock Holmes: mind wandering increases with perceptual processing difficulty during reading and listening

**DOI:** 10.1186/s41235-023-00483-0

**Published:** 2023-05-25

**Authors:** Lena Steindorf, Sebastian Pink, Jan Rummel, Jonathan Smallwood

**Affiliations:** 1 Department of Psychology, Heidelberg University, Hauptstrasse 47-51, 69117 Heidelberg, Germany; 2 Mannheim Centre for European Social Research, University of Mannheim, Mannheim, Germany; 3 Department of Psychology, Queen’s University, Kingston, ON Canada

**Keywords:** Mind wandering, Task-unrelated thought, Perceptual processing difficulty, Text comprehension

## Abstract

We investigated whether increased perceptual processing difficulty during reading or listening to a Sherlock Holmes novella impacts mind wandering as well as text comprehension. We presented 175 participants with a novella in either a visual or an auditory presentation format and probed their thoughts and motivational states from time to time during reading/listening. For half of the participants in each presentation-format condition (visual or auditory), the story was superimposed by Gaussian noise. For both presentation formats, the participants who were exposed to noise while processing the story mind-wandered more and performed worse in a later comprehension test than the participants who processed the story without added noise. These negative effects of increased perceptual processing difficulty on task focus and comprehension were partly driven by motivational factors: reading/listening motivation mediated the relationship between perceptual processing difficulty and mind wandering.

## Introduction

The abilities to read and listen are key methods through which we gather information in our everyday lives. People read the newspaper in the morning, listen to a colleague at work, and read the menu to decide what to eat for lunch. Sometimes, however, the to-be gathered information is not presented in a clear and resolvable manner, as examples such as background noise, illegible handwriting, or connection problems during video conferencing demonstrate. Arguably, in such situations, reading or listening tasks become more difficult to process because the perceptual information from which meaning is derived is compromised. In the current work, we tested whether increased *perceptual processing difficulty* (hereinafter, perception difficulty) would impact people’s capability to focus their attention on a reading or listening task and, in turn, their comprehension performance. Importantly, from previous research, it is not clear whether increased perception difficulty should positively or negatively affect attention. Somewhat counterintuitively, it has previously been shown that making perceptual processing of task stimuli more difficult can lower people’s likelihood to mind-wander and increase task performance (Faber et al., 2017; Forster & Lavie, 2009). However, reduced readability or audibility during narrative comprehension might also lead to opposite effects, that is increased mind-wandering rates and decreased task performance. Evidence from recent mind-wandering research in the domain of reading, that manipulated processing difficulty on a semantic level (e.g., via syntactic complexity), is consistent with this assumption. Reading semantically difficult in comparison to easy texts is often associated with more drifting thoughts and worse text comprehension (e.g., D'Mello & Mills, 2021; Feng et al., 2013; Kahmann et al., 2021; Smallwood, 2011).

Our study also set out to understand whether the modality of presentation plays an important role in mind wandering and comprehension. Most studies reviewed in the present work employed visually to-be-processed tasks. Little research has been conducted investigating mind wandering during listening tasks (but see Konu et al., 2021), and none of these studies investigated effects of perception difficulty. Similarly, there is not yet a clear picture concerning the effects of presentation modality on comprehension variables (Rogowsky et al., 2016), especially when it comes to the effect of perception noise. To aid in closing this research gap, we included a listening task in addition to commonly used reading tasks in our experiment. For the present work, we assumed that listening and reading share similar processes necessary for extracting meaning from sensory input (Gernsbacher et al., 1990), and so did not predict specific group differences for attention and comprehension measures between reading and listening groups. In summary, our aim was to examine whether and in which direction perception difficulty influences mind wandering and comprehension when people read or listen to a short crime novel.

## Mind wandering

Across a range of different tasks, remaining focused is a necessary prerequisite for successful task completion, for example, when trying to comprehend the plot of a crime novel. However, several forms of distractors are omnipresent, possibly endangering text comprehension. Such distractors can be external (e.g., a passing fire truck with blue lights and sirens in operation), but—important for the present work—also self-generated and internal, such as wandering or task-unrelated thoughts (Seli et al., 2018; Smallwood & Schooler, 2015), which people experience to a high level in everyday life (Kane et al., 2017; Killingsworth & Gilbert, 2010). One goal of research into mind wandering is to identify factors that reduce or increase the susceptibility to internal distractors. Such factors may lie within a person or the current environment (e.g., hunger, mood, or stress, see Engert et al., 2014; Rummel & Nied, 2017; Smallwood et al., 2009), or the ongoing task itself (e.g., task difficulty, see Kahmann et al., 2021; Rummel & Boywitt, 2014). The current work focuses on the factor of perception difficulty (reduced readability/audibility due to visual/auditory noise), which concerns the task itself and might influence mind-wandering rates in two directions.

### Resource-competition assumption

On the one hand, one could assume that mind-wandering rates during reading or listening decrease with increased perception difficulty. This claim is based on the idea of a limited pool of attentional resources that is shared by task and distractor processing. Increasing a task’s perceptual demands might bind attention to the task itself thereby leaving less resources available for the processing of external as well as internal distractors such as mind wandering (Faber et al., 2017; Forster & Lavie, 2009). In line with this idea of task-focus benefits due to increased perception difficulty, mind wandering was found to be less frequent under high perceptual-demand conditions in a visual-search task (Forster & Lavie, 2009). Further, Faber et al. (2017) found that less mind wandering was reported when participants read a text in a disfluent as compared to a regular font. They argued that reading a text written in gray and in the Comic Sans font requires more attentional resources than reading one in black and the Arial font, leaving fewer resources available for mind-wandering processes. Accordingly, as evident from our preregistration of the present study (https://osf.io/6ry5h), we hypothesized that beneficial effects regarding the participants’ task focus could emerge in conditions with reduced readability and audibility.

### Overload assumption

On the other hand, one could assume that mind-wandering rates during reading or listening increase with increased perception difficulty due to the rather complex nature of reading and listening tasks. It has been argued that successful task engagement and hence text comprehension require the reader to stay focused for long periods of time to be able to connect different text passages as well as prior knowledge and to eventually create a *situational model* of the storyline (Feng et al., 2013; Gernsbacher et al., 1990; Kintsch & van Dijk, 1978; Smallwood, 2011; Smallwood et al., 2008). Kahmann et al. (2021) described such a model as “an extensive mental representation of the meaning of the concepts and events described in the text, their implied context, and their connection to pre-existing knowledge” (p. 2). Reading and listening are thus assumed to constitute complex higher-level cognitive tasks (Feng et al., 2013; Gernsbacher et al., 1990; Kintsch & van Dijk, 1978), during which comprehension activities that make use of and simultaneously form a situational model should always be maintained. This process not only requires continual external attentional focus but also aids the comprehension process: Once formed, a situational model can help direct the reader to information within the narrative that is important for comprehension, thus benefitting efficient attention allocation. For example, in a study by Smallwood and colleagues (2008), readers of a crime novel who had an intact situation model read with focused attention at the moments when important clues concerning the identity of the villain were given. Readers who were not as successful at building a situation model, however, often zoned out at these most important parts of the story.

When the information that is used to construct a situational model become degraded, it may compromise the individual’s ability to create the model, denying them the chance to benefit from the model itself. Increasing any kind of processing difficulty within the already complex and resource-demanding tasks of reading and listening may thus lead to overload and corresponding costs like text disengagement and increased mind-wandering rates.

Consequently, situational-model building should be more likely to fail in specific situations, such as when reading or listening to texts with increased difficulty, for example, at the semantic level. While reading texts with a highly complex syntax and a multitude of specialist or unfamiliar terms, engagement with distractors like wandering thoughts should become more likely. And indeed, current findings seem to be in line with this reasoning: When reading difficult compared to easy texts, mind-wandering rates have repeatedly been found to increase (Feng et al., 2013; Kahmann et al., 2021; Mills et al., 2015; Soemer & Schiefele, 2019; Soemer et al., 2019)^1^ and this increase seems to follow a linear trend (Kahmann et al., 2021). However, it remains an open question whether such costs regarding the participants’ task focus might also be present when it comes to perception difficulty, which would contradict the idea of task-focus benefits due to attention-binding processes, which we proposed in our preregistration.

## Motivational variables

Particularly in the light of the overload assumption, we propose that motivational variables will play an important role because difficulties with building a situational model are likely to be accompanied by motivational declines.

Increased processing difficulty should render text comprehension more cognitively demanding (Kahmann et al., 2021). Referring to extended models of motivation-cognition interactions (e.g., Kool et al., 2010) and cost–benefit analyses, Kahmann and colleagues (2021) argued that a reader might feel that further focused reading is not “worth it” when processing is overly difficult. The increased cognitive effort (i.e., costs) might feel rather aversive and might not be accompanied by adequate benefits, leading the reader to escape into currently more rewarding mental content (i.e., mind wandering). This process could be enhanced when situational-model building is actually impaired or fails, given that the benefits of further focused reading (comprehending the text, learning something new, etc.) become even less likely.^2^ Even though Kahmann and colleagues focused on semantic processing difficulty, this rationale may translate to perceptual processing difficulty which can also be experienced as cognitively exhausting (Lavie & Tsal, 1994).

Motivation to perform well on a task has been shown to be a driving force for keeping mind wandering at a low level (e.g., Seli et al., 2019; Unsworth & McMillan, 2013). When, during narrative comprehension, motivation is lowered due to both high cognitive demands and/or impaired situational-model building, mind wandering is likely to increase. Consistent with this assumption, Kahmann (2021) as well as Soemer and Schiefele (2019) found topic interest to be reduced and task-unrelated thoughts to occur more frequently when reading became semantically more difficult. In the current work, we tested for a decline in reading and listening motivation due to increased *perception* difficulty, which should, in turn, go along with higher mind-wandering rates (Seli et al., 2019; Unsworth & McMillan, 2013).

Therefore, we included exhaustion and motivation items as proxy indicators of increased cognitive effort, aversiveness, and motivational declines that we expect to emerge due to overload processes initiated by increased perception difficulty. With regard to the opposing attentional-resources-competition assumption, we did not have specific predictions concerning these motivational variables.

## Text comprehension

Mind wandering has repeatedly been found to increase the risk of text-comprehension failures (e.g., Feng et al., 2013; Steindorf & Rummel, 2020). Due to this negative relationship between the amount of task-unrelated thoughts and comprehension performance, increased perception difficulty could hamper or foster reading/listening comprehension, depending on which of our two assumptions above (resource-competition versus overload assumption) passes the empirical test. Similar to Smallwood et al. (2008), we were interested in *fact-based* and *inference-based* comprehension. This way, we could differentiate between mind wandering impairing mere knowledge about story details (fact-based) on the one hand, and the construction of inferences (inference-based) on the other hand. Inference-based comprehension goes beyond pure fact knowledge because several story events and sometimes even previous knowledge have to be integrated in order to draw conclusions about, for example, the identity of a crime-novel villain. As perception difficulties occur early on during task processing, we assumed that a high level of perception difficulty should affect both fact-based and inference-based comprehension performance to some extent.

## The present study

The present study aimed to understand how perception difficulty is linked to task-focus benefits or costs (i.e., less or more mind wandering and better or worse text comprehension) during narrative comprehension, considering both verbal and auditory modalities. To do so, we presented one half of participants with a short Sherlock Holmes novel without additional perceptual noise (low perception difficulty), whereas the other half received a version of the same story, for which the text was superimposed by Gaussian noise (high perception difficulty). The participants were presented with the text in either a visual or an auditory presentation format. We sampled the participants’ thoughts and motivational states during reading and listening and assessed their text comprehension at the end. Visual perception difficulty (hereinafter, visual difficulty) during reading was imposed by adding gray Gaussian noise to the background, auditory perception difficulty (hereinafter, auditory difficulty) by adding auditory Gaussian noise to the sound file.

We chose this specific operationalization of perception difficulty because it represents an arguably stronger manipulation than the one employed by Faber and colleagues (2017). Their participants did not subjectively perceive the reading task to be more difficult or effortful when the text was made more perceptually difficult (by using an unfamiliar font printed in gray on a white background). A stronger manipulation may thus trigger problems with situational-model building which were not present in Faber et al.’s study. Our manipulation is of high external validity as, in everyday life, perception difficulty is not always as subtle as a text being written in an unfamiliar font, but can often reach the level of almost illegible writing or nearly completely distorted audio channels.

In the following Method and Results sections, we report how we determined our sample size and all data exclusions, as well as all measures in the study (Simmons et al., 2012). Our data set (https://osf.io/f5rgp) and analyses script (https://osf.io/u5xv6) can be found on our OSF project page.

## Methods

### Data collection, participants, and design

Because of the COVID-19 pandemic, we collected data online using the Qualtrics software (Qualtrics, Provo, UT, https://www.qualtrics.com) from February 24 to March 24, 2021. 220 participants finished the experiment (as preregistered). As we intended to run multilevel regression analyses to test our main research questions, we based our planned sample size on recommendations from simulation studies by Maas and Hox (2005). 45 participants were excluded from the analyses. Exclusions were performed due to participants reporting serious technical difficulties (such as connection problems, *n* = 6), serious disturbances (such as longer phone calls or leaving the room for an extended period, *n* = 5), or not having provided honest information (*n* = 3) on control questions at the end of the experiment. We further excluded those who took more than four hours to complete the survey (*n* = 6) and those who had already been familiar with the story before taking part in the experiment (*n* = 25). This resulted in a final sample size of *N* = 175 (*M*_age_ = 24.14, *SD*_age_ = 6.36; 139 female, 33 male, 3 without gender disclosure, 159 German native speakers, and 169 with secondary school education or higher).

We employed a two-factorial design with both the perception-difficulty level (no-noise versus noise) and the presentation format (visual versus auditory) manipulated between participants. The participants were randomly assigned to the four groups: visual presentation without noise (*n* = 46), visual presentation with noise (*n* = 48), auditory presentation without noise (*n* = 42), and auditory presentation with noise (*n* = 39).

### Materials

#### Reading/listening material and text-comprehension assessment

As the reading and listening material we used a German version of the short crime novella *The Red-Headed League* (Conan-Doyle, 2001). This story has been used before (in the original English version) by Smallwood and colleagues (2008) to examine mind wandering during reading. The text was edited so that it excluded the introduction segment as well as the segment in which the crime is solved and contained roughly 5,500 words (comparable to Smallwood et al., 2008).

**Fig. 1:**
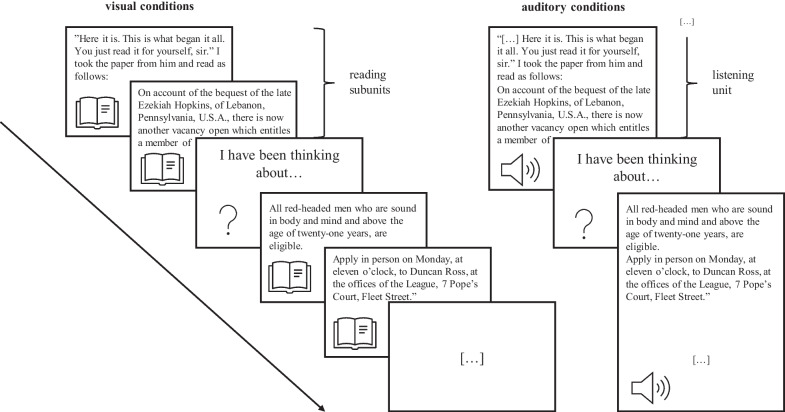
Story-presentation and thought-probing procedure. *Note* In both (noise and no-noise) visual conditions, the participants were presented with the novella’s written text subunit by subunit until a thought probe appeared. In both (noise and no-noise) auditory conditions, the participants were presented with the audio recording of the novella with each listening unit being followed by a thought probe. After a thought probe, in all conditions, the story presentation continued. It is apparent that each listening unit comprised several reading subunits and thought probes appeared at the same text positions for all conditions

The participants in the two conditions with an auditory presentation format were presented with an audio file of the novella that was recorded by a female German native speaker. The audio recording had a total length of 33 min and was presented to the participants within 19 successive units (*M*_presentation time/unit_ = 104.40 s, *SD*_presentation time/unit_ = 55.53 s), which were each (except for the last one) followed by a thought probe (see below and Fig. 1). While listening to the text, the participants were presented with a blank screen.

In both visual conditions, we presented the novella on the computer screen in black Arial font on a white background. The text had an easily-readable size, which was kept constant across various devices.^3^ To create units for the text presentation, we further subdivided the text of each of the 19 *listening units* into several *reading subunits*. We ended up with 187 subunits in total (*M*_word count/subunit_ = 29.20, *SD*_word count/subunit_ = 7.24), which were presented to the participants successively. This subdivision allowed for thought probes to appear at unpredictable time points as well as at the same text positions for all conditions, namely when a listening unit or the respective reading subunit ended (see Fig. 1). To align the presentation times for the written and the audio-recorded materials, we extracted the listening times of the reading subunits from the audio recording and used these times to determine the presentation duration of the reading subunits.

To assess reading and listening comprehension, we first created 32 multiple-choice questions concerning the novella with four response options each (always one correct and three incorrect options). These questions were then piloted on 20 participants (*M*_age_ = 34.25, *SD*_age_ = 17.84; 11 female, 18 German native speakers) who did not take part in the main experiment. Based on the pilot data, we chose 14 questions to include in the main experiment considering their difficulty (*M*_difficulty_ = 80.36%, *SD*_difficulty_ = 15.12%) but also their position within the storyline: The events that twelve of the chosen questions referred to were distributed approximately evenly across the story (fact-based questions, e.g., “Where did Holmes, Watson, Mr. Jones, and Mr. Merryweather meet in the evening at 10 pm?”). Two final comprehension questions did not refer to a specific time point, but relied on the integration of many story events asking for the identity of the villain (see Smallwood et al., 2008) and the storyteller (inference-based questions). The internal consistency of the whole questionnaire was 0.77 (McDonald’s ω), which can be considered acceptable (Catalán, 2019).

#### Manipulation of perception difficulty

**Fig. 2:**
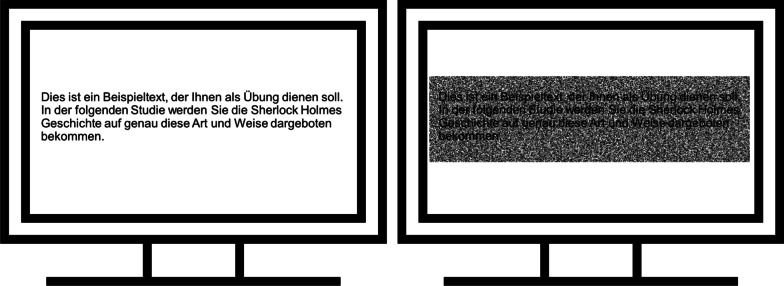
Manipulation of visual difficulty*. Note* The left part of the figure shows how the text appeared in the no-visual-noise condition, whereas the right part shows the text within the visual-noise condition. The text used in the figure is a passage of German instructional text which was used as part of a practice trial at the beginning of the study

In the condition with high visual perception difficulty, we masked the novella’s written text with static Gaussian visual background noise (for a similar approach, see Hughes et al., 2013). We added monochromatic Gaussian noise (set to the maximum of 400% in Adobe Photoshop CC 2020) to a white canvas and converted the resulting grayscale to Bitmap using a 50% threshold, generating a black-and-white image with a resolution of 72 pixels per inch. Figure 2 illustrates the visual difficulty manipulation.

In the condition with high auditory difficulty, we used continuous Gaussian white noise with a flat volume spectrum over the range of the audible frequencies of the recorded female voice. To both assert this fit and to prevent technical problems with playing very low and very high frequencies (frequencies that tend to be exacerbated by standard audio devices), we cut off frequencies below 250 Hz with a steep 48 dB (per octave) slope as well as all frequencies above 2 kHz with a flatter 12 dB slope out of the Gaussian white noise. We set the noise’ volume to − 5 dB which corresponds to the average volume of the female voice. An example of the auditory difficulty can be found on the OSF (no-noise example: https://osf.io/zvnhp; noise example: https://osf.io/xd4k3).

#### Mind wandering conceptualization and assessment

We conceptualized mind wandering as task-unrelated thoughts for the present study (Seli et al., 2018) and explained the concept to our participants accordingly, including examples and the affirmation that mind wandering is a naturally occurring phenomenon that happens to everyone. We used the thought-probing method (Weinstein, 2018) to assess mind wandering by asking the participants about their current thoughts 18 times during reading or listening. Answering options were [1] *I am thinking about the Sherlock Holmes story* and [2] *I am thinking about something unrelated to the Sherlock Holmes story.*^4^ The positions of twelve thought probes within the story were aligned with events that comprehension questions referred to (e.g., a thought probe appeared after the story characters met at a certain destination, and the later comprehension question asked where the characters met). Six more thought probes were added to assure an even distribution of thought probes across the entire story.

#### Assessment of motivational factors

At the beginning, in the middle, and at the end of the story, all participants were asked how motivated they were to follow the storyline (on a rating scale of 0 to 10 ranging from “*not motivated at all*” to “*very motivated*”) and how exhausted they currently felt (on a rating scale of 0 to 10 ranging from “*not exhausted at all*” to “*very exhausted*”).

#### Manipulation-check and control questions

Two manipulation-check items assessed whether factors within the study (“*Certain factors within the study [e.g., the presentation form of the story] made it difficult for me to follow the story.*”) or outside of the study (“*Certain factors outside of the study [e.g., my smartphone or other people] made it difficult for me to follow the story.*”) hampered the participants’ comprehension. Both items were answered on a rating scale of 1 to 5 ranging from “I *do not agree at all*” to “*I fully agree*.” Further control questions asked the participants to indicate whether they experienced serious technical difficulties (such as connection problems) or serious disturbances (such as longer phone calls or leaving the room for an extended period), whether they provided honest information in the course of the study, and whether they had been familiar with the novella before taking part in the experiment.

### Procedure

First, the participants gave informed consent, started the full-screen mode on their device, and confirmed that they were working on the study without distraction, before randomly being assigned to one of the four experimental conditions. They were then presented with condition-specific instructions concerning the reading or listening task as well as mind-wandering instructions. In both auditory conditions, the participants were asked to put on headphones before listening to a short instructional text with or without noise (depending on their condition) to make sure that their audio worked and was set to a pleasant volume as well as to familiarize them with the presentation format. Then, they were asked not to change their volume settings during the remainder of the study. Similarly, participants in both visual conditions were asked to adjust their monitor’s brightness to a comfortable setting during the presentation of seven subunits of instruction text (same text as in the auditory conditions) with or without noise (depending on their condition) and not to change it at a later time point in the study.

Following this practice phase, all participants were presented with the novella including thought probes. Participants in the visual conditions read the story experimenter paced, subunit by subunit. Unnoticeable for participants, several subunits formed a reading unit, resulting in 19 units in total (equivalent to the listening units). Eighteen thought probes were presented, one after each unit expect for the last one. Analogously, in the auditory conditions, 19 listening units were presented one after the other and separated from each other by 18 thought probes. Furthermore, at the beginning, in the middle, and at the end of the story, the motivational factors were assessed. After being presented with the story, participants were asked to answer the comprehension questions. Then, they filled out two questionnaires^5^ before answering the manipulation-check and control questions concerning their experience and behavior during the study. Finally, participants answered sociodemographic questions before being debriefed and dismissed.

## Results

### Manipulation check

**Fig. 3:**
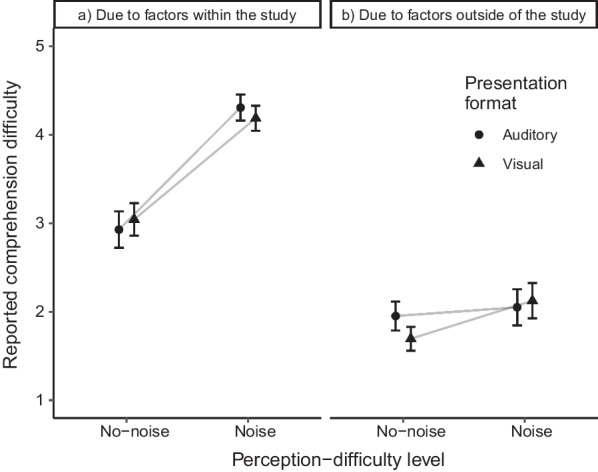
Results of the manipulation check. *Note* As apparent from the left part of the figure (**a**), the manipulation check revealed a heightened difficulty during reading/listening due to factors within the study for participants in the noise conditions. There was no statistical evidence for presentation-format group differences. The right panel (**b**) illustrates that there was no statistical evidence for any group differences concerning the perceived difficulty due to factors outside of the study. Error bars depict the standard errors of the means

To test whether increased visual or auditory perception difficulty affected the perceived comprehension difficulty and whether this was only due to factors within (e.g., the presentation form of the story) and not outside of the study (e.g., their smartphone or other people in the room), we ran two analyses of variance ((Bayesian) ANOVAs, see Fig. 3) using JASP (JASP Team, 2022). Because we sought evidence for differences as well as comparability between groups within these analyses, we report Bayes factors in addition to the information for frequentist ANOVAs. More specifically, we either report BF_10_ as the Bayes factor for the alternative versus the null hypothesis, or BF_01_ as the Bayes factor for the null versus the alternative hypothesis.^6^ For all reported Bayesian analyses in the Results section, JASP’s default prior option (Cauchy priors) was selected.

We first ran a 2 × 2 (Bayesian) ANOVA with the perception-difficulty level (no-noise versus noise) and the presentation format (visual versus auditory) as between-participants factors and the perceived difficulty due to factors *within* the study as the dependent variable. The ANOVA revealed a significant difference between the no-noise and the noise conditions, *F*(1,171) = 53.70, *p* < 0.001, η_p_^2^ = 0.24. Concerning this main effect of the perception-difficulty level, the corresponding BANOVA further provided decisive support in favor of the alternative hypothesis, BF_10_ > 100. As apparent from Fig. 3a, the participants in the noise conditions perceived the task as more difficult. The main effect of the presentation format, *F*(1,171) < 1.00, *p* = 0.998, η_p_^2^ < 0.01, and the interaction, *F*(1,171) < 1.00, *p* = 0.496, η_p_^2^ < 0.01, were not significant. In addition, the corresponding Bayes factors of BF_01_ = 7.48 (main effect of the presentation format) and BF_01_ = 7.11 (interaction) substantially supported the null hypothesis.

For the perceived comprehension difficulty due to factors *outside* of the study (Fig. 3b), an ANOVA with the same factors revealed no significant main effect for neither the perception-difficulty level, *F*(1,171) = 2.21, *p* = 0.139, η_p_^2^ = 0.01, nor the presentation format, *F*(1,171) < 1.00, *p* = 0.607, η_p_^2^ < 0.01. Additionally, the corresponding Bayes factors of BF_01_ = 2.86 (for the perception-difficulty level, anecdotal support) and BF_01_ = 7.36 (for the presentation format, substantial support) provided support in favor of the null hypothesis. The interaction of the two factors was also not significant, *F*(1,171) < 1.00, *p* = 0.354, η_p_^2^ < 0.01, with the null hypothesis being strongly supported by the Bayes factor, BF_01_ = 15.06. This pattern suggests that participants of all conditions reported, on average, similar levels of perceived comprehension difficulty due to factors outside of the study.

### Descriptive data and correlations

As apparent from Table 1, the probability to mind wander during both reading and listening was descriptively higher in the noise conditions. The participants in the noise conditions further performed worse in the comprehension test, and this applied to both fact-based and inference-based questions. These differences between the no-noise and noise conditions concerning mind wandering and comprehension are descriptively present for both presentation formats.

**Table 1:** Means and standard deviations (in parentheses) for the mind-wandering and comprehension variables, separately for each experimental condition

Variable	Visual presentation format		Auditory presentation format	
	No-noise	Noise	No-noise	Noise
Mind wandering [%]	15.82 (13.71)	21.53 (22.18)	18.65 (17.56)	27.92 (23.28)
Fact-based comprehension [0–12]	9.98 (1.98)	8.65 (2.36)	9.07 (2.34)	7.74 (2.71)
Inference-based comprehension [0–2]	1.52 (0.66)	1.25 (0.73)	1.36 (0.66)	1.13 (0.70)

Values in brackets indicate the scale of the measure. Mind-wandering values were aggregated across all 18 assessment points. Values for fact-based comprehension are based on the twelve fact-based questions that refer to specific events distributed across the whole story. Values for inference-based comprehension are based on the two inference-based questions that require the integration of many story events

As expected, mind wandering was negatively correlated with comprehension (Table 2). Overall, participants who mind-wandered more, performed worse when answering fact-based as well as inference-based comprehension questions. Mind wandering was also negatively associated with reading/listening motivation and positively with exhaustion. Interestingly, these relations are descriptively strongest for the motivation and exhaustion reports that were assessed in the middle and at the end of the story. Separate correlation tables for each experimental condition can be found on our OSF page (https://osf.io/8ntxu).

**Table 2:** Pearson correlations across conditions between the mind-wandering and comprehension variables and with the motivational variables

	Mind wandering	Fact-based comprehension	Inference-based comprehension
Mind wandering	–		
Fact-based comprehension	− 0.58***	–	
Inference-based comprehension	− 0.27***	0.48***	–
Motivation (beginning)	− 0.17*	0.07	0.04
Motivation (middle)	− 0.55***	0.44***	0.21**
Motivation (end)	− 0.64***	0.49***	0.27***
Exhaustion (beginning)	0.13	0.03	0.12
Exhaustion (middle)	0.40***	− 0.20**	− 0.01
Exhaustion (end)	0.38***	− 0.22**	− 0.06

All values were aggregated across all experimental conditions. Mind-wandering values were further aggregated across all assessment points. Motivational values (motivation and exhaustion) are displayed separately for the three assessment points at the beginning, in the middle, and at the end of the story. For inference-based comprehension (a three-level variable), Spearman’s Rho correlations were almost identical to the reported Pearson correlations, both in magnitude and *p* values

^***^p < 0.001; **p < 0.01; *p < 0.05

### Mind wandering

To statistically test the descriptively present costs of noise on the mind-wandering variable, we ran multilevel analyses (cf. Steindorf & Rummel, 2020). As preregistered, we regressed mind-wandering-probe responses that were repeatedly assessed per person, on the perception-difficulty level, the presentation format, and the time on task using a generalized linear model with a logistic link function estimating fixed effects for all predictors and random intercepts for each participant (using the lme4 package in R, Bates et al., 2015). Mind wandering entered the analysis as a binary variable (0 = no mind wandering/task focus, 1 = mind wandering) of which 18 assessment points were nested in participants. The effects of the perception-difficulty level (0 = no-noise, 1 = noise) and the presentation format (0 = auditory, 1 = visual) were estimated based on binary variables. Time on task was entered as a metric variable that indicated the sequence of thought probes (i.e., 0, 1, …, 17) embedded in the novella.

The results of this multilevel regression analysis are shown in the Model-A-panel of Table 3. They confirm the descriptive trend of negative effects of noise on the participants’ task focus: The occurrence of mind wandering was more likely in the noise conditions than in the no-noise conditions. Further, there was no statistical evidence for the presentation format influencing the mind-wandering likelihood. However, the latter increased with the time that was spent on the task.

**Table 3:** Results of the multilevel models investigating mind wandering, fact-based comprehension and inference-based comprehension

Model	Criterion	Predictor	*b*	*SE*_*b*_	Wald *Z*	*p*
A	Mind wandering	Perception-difficulty level	0.48	0.21	2.26	0.024
		Presentation format	− 0.36	0.21	− 1.68	0.094
		Time on task	0.04	0.01	4.32	0.001
B1	Fact-based comprehension	Perception-difficulty level	− 0.68	0.18	− 3.87	0.001
		Presentation format	0.46	0.18	2.63	0.009
		Time on task	− 0.05	0.02	− 3.41	0.001
B2	Fact-based comprehension	Perception-difficulty level	− 0.61	0.16	− 3.78	0.001
		Presentation format	0.43	0.16	2.64	0.008
		Time on task	− 0.04	0.02	− 2.57	0.010
		Mind wandering	− 0.88	0.14	− 6.45	0.001
C1	Inference-based comprehension	Perception-difficulty level	− 0.71	0.29	− 2.42	0.015
		Presentation format	0.45	0.29	1.54	0.124
C2	Inference-based comprehension	Perception-difficulty level	− 0.55	0.30	− 1.82	0.069
		Presentation format	0.34	0.30	1.16	0.247
		Mind wandering	− 0.14	0.04	− 3.17	0.002

Model A denotes a generalized linear model (logistic link function) predicting 18 mind-wandering-assessment points by the perception-difficulty level, the presentation format and the time on task using fixed covariate effects and random intercepts. Model B denotes a generalized linear model (logistic link function) predicting twelve assessment points of fact-based comprehension by the perception-difficulty level (Models B1 and B2), the presentation format (Models B1 and B2), the time on task (Models B1 and B2), and twelve mind-wandering-assessment points (corresponding to the question events in terms of assessment time, Model B2) using fixed covariate effects and random intercepts. Model C denotes an ordered regression model (logistic link function) predicting three levels of inference-based comprehension by the perception-difficulty level (Models C1 and C2), the presentation format (Models C1 and C2), and mind wandering (the sum of all mind-wandering instances during all 18 assessment points, Model C2). Please find the information of the variable coding in the running text. *SE* standard error

### Comprehension performance

As negative effects of noise should not only be reflected on the mind-wandering variable, but also by worse comprehension performance under noise conditions, we ran further analyses using fact-based and inference-based comprehension as dependent variables. For fact-based comprehension, as preregistered, we first (Model B1, Table 3) regressed the participants’ accuracy on each of the twelve fact-based comprehension questions (questions nested in participants, 0 = incorrect response, 1 = correct response) on the perception-difficulty level (0 = no-noise, 1 = noise), the presentation format (0 = auditory, 1 = visual), and the time on task (metric variable indicating the sequence of question events within the novella, i.e., 1, 2, …, 12) using a generalized linear model with a logistic link function estimating fixed effects for all predictors and random intercepts for each participant (using the lme4 package in R, Bates et al., 2015). In a second step, we additionally included mind wandering in this model (Model B2, Table 3). Mind wandering was operationalized as the indicated thought mode (0 = task focus, 1 = mind wandering) on the twelve thought probes that temporally corresponded to the events, which the fact-based comprehension questions referred to, and was thus also nested within participants.

As apparent from Models B1 and B2 in Table 3, all predictors significantly predicted fact-based comprehension. The probability to give a correct answer significantly decreased for the participants in the noise conditions mirroring the descriptively present costs of noise on the fact-based-comprehension variable. Interestingly, mind wandering, which we previously found to be predicted by the perception-difficulty level, also negatively predicted fact-based comprehension (Model B2). This indicated that both, mind wandering and the perception-difficulty level, contributed to the prediction of fact-based comprehension above and beyond the effect of the respective other. Further, visual presentation in comparison with auditory presentation increased the probability to answer fact-based comprehension questions correctly. Finally, the time-on-task variable significantly negatively predicted fact-based comprehension.

For the criterion of inferenced-based comprehension, we employed ordered (logistic) regression models (Fullerton, 2009) using the MASS package in R (Venables & Ripley, 2013) because participants’ answers were restricted to three values, namely zero, one, and two correct answers. We considered the time-on-task predictor from the previous analyses as not meaningful in the Models C1 and C2 (Table 3), as the inferenced-based questions were independent of the story’s time course and rather required the conceptualization of the story as a whole. We first (Model C1, Table 3) regressed the inference-based comprehension on the perception-difficulty level (0 = no-noise, 1 = noise) and the presentation format (0 = auditory, 1 = visual). The probability to give a correct answer significantly decreased for the participants in the noise conditions,^7^ but had no statistically significant relationship with the presentation format. In a second step (Model C2, Table 3) that additionally included the absolute frequency of mind-wandering instances observed per participant during the comprehension task (i.e., up to 18) as a predictor, the previously significant effect of perception difficulty vanished while mind wandering significantly and negatively predicted inference-based comprehension.

### Motivational factors and mediation model

**Fig. 4:**
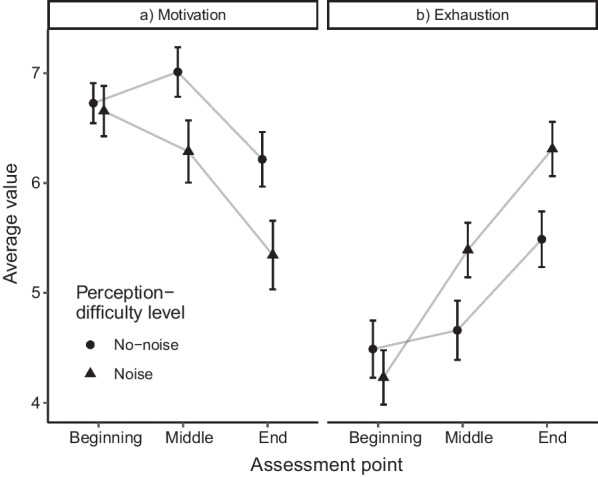
Motivation and exhaustion in the course of time. *Note* The figure shows average values of **a** the motivation and **b** the exhaustion variables assessed at the beginning, the middle, and the end of the novella, separately for the noise and no-noise conditions. The scale ranges from 0 to 10 for both dependent variables. The displayed values were aggregated across both presentation formats (visual and auditory), because this factor did not interact with the time course. Error bars depict standard errors of the means

To examine the influence of the motivational factors on the observed task-focus and comprehension costs due to noise, we ran further not preregistered exploratory analyses. First, we examined the time course of motivation and exhaustion (Fig. 4). For these analyses, we report Bayes factors in addition to the information for frequentist ANOVAs, as we did for the manipulation-check analyses. Taking into account that the experimental groups might differ on some assessment points but not on others (e.g., not on the very first assessment), we considered both BF_10_ (as the Bayes factor in favor of the alternative hypothesis) and BF_01_ (as the Bayes factor in favor of the null hypothesis) to be informative for the following analyses.

We employed a 3 × 2 × 2 mixed (Bayesian) ANOVA with the assessment point (before, in the middle of, and at the end of the reading phase) as a within-participants factor and the perception-difficulty level (no-noise versus noise) as well as the presentation format (visual versus auditory) as between-participants factors for the current reading/listening motivation as the dependent variable (Fig. 4a). This analysis was carried out in JASP (JASP Team, 2022). The (B)ANOVA revealed a significant main effect of the assessment point, *F*(2, 342) = 17.01, *p* < 0.001, η_p_^2^ = 0.03, with the corresponding Bayes factor providing decisive support for the alternative hypothesis, BF_10_ > 100. The interaction between the assessment point and the perception-difficulty level was significant, *F*(2, 342) = 3.18, *p* = 0.043, η_p_^2^ = 0.01, but the Bayesian analysis suggested no support for the alternative hypothesis, BF_10_ = 0.56. All other main effects and interactions remained non-significant, all *F*s < 3.68, all *p*s > 0.057, with Bayes factors providing at least anecdotal support for the null hypotheses, all BFs_01_ > 1.72. Post hoc tests indicated that there was no significant motivation difference between the noise and the no-noise conditions before the reading/listening phase, *t*(173) < 1.00, *p* = 0.805, *d* = 0.04, with the null hypothesis being substantially supported by the corresponding Bayes factor, BF_01_ = 5.94. However, in the middle of, *t*(173) = 2.00, *p* = 0.047, *d* = 0.30, BF_10_ = 1.04, and at the end of the story, *t*(173) = 2.18, *p* = 0.030, *d* = 0.33, BF_10_ = 1.47, the groups significantly differed in terms of the reported motivation levels with lower values having been reported in the noise conditions. Additionally, corresponding Bayes factors provided anecdotal support for the alternative hypotheses.

Analogously to the ANOVA for motivation, we analyzed the time course of the reported exhaustion (Fig. 4b). There was a significant main effect of the assessment point, *F*(2, 342) = 61.19, *p* < 0.001, η_p_^2^ = 0.07, as well as a significant interaction between the assessment point and the perception-difficulty level, *F*(2, 342) = 9.11, *p* < 0.001, η_p_^2^ = 0.01, with the corresponding Bayes factors additionally providing decisive support for the alternative hypotheses, both BFs_10_ > 100. The Bayesian analysis suggested that the significant main effect of the presentation format was anecdotal, *F*(1, 171) = 3.91, *p* = 0.050, η_p_^2^ = 0.02, BF_10_ = 0.70. Numerically, participants in the auditory conditions (*M* = 5.43, *SD* = 1.92) were more exhausted than those in the visual conditions (*M* = 4.80, *SD* = 2.28). All other main effects and interactions remained non-significant, all *F*s < 1.95, all *p*s > 0.165, with Bayes factors providing at least anecdotal support for the null hypotheses, all BFs_01_ > 2.31. We broke down the interaction between the perception-difficulty level and the assessment point and found no significant exhaustion difference between the noise and the no-noise conditions before the reading/listening task started, *t*(173) = 0.72, *p* = 0.472, *d* = 0.11. In addition, the corresponding Bayes factor provided substantial support in favor of the null hypothesis, BF_01_ = 4.80. In the middle of the task, *t*(173) =  − 2.00, *p* = 0.048, *d* =  − 0.30, BF_10_ = 1.03, and at the end of the task, *t*(173) =  − 3.22, *p* = 0.022, *d* =  − 0.35, BF_10_ = 1.94, however, exhaustion tended to be stronger in the noise than the no-noise conditions.

In sum, we found motivation as well as exhaustion to be influenced by perception difficulty, but only after participants had been able to actually experience what reading/listening to the story under noise or no-noise conditions would be like (see second and third assessment points). The absence of a statistically significant condition difference at the first assessment point (i.e., before the task started) that was substantially supported by the Bayes factors can be interpreted as a demonstration that the randomized assignment of participants to experimental groups was successful. It is, however, not indicative of perception-difficulty effects on motivational variables. Because differences in the motivational variables emerged at the second and third assessment point, we used the values assessed at these points when specifying a path model describing possible mental and motivational processes that eventually result in a negative relationship between perception difficulty and reading comprehension.

Using the lavaan package (Rosseel, 2012) in R, we specified a similar structural path model as Soemer and Schiefele (2019), who aimed to explain the relationship between semantic processing difficulty and reading comprehension. Perception difficulty, motivation and exhaustion were modeled as manifest variables. Mind wandering and comprehension were modeled as latent variables. All variables were aggregated across both presentation formats (visual and auditory), because we did not assume different mediating processes for the varying presentation modalities. The perception-difficulty variable constituted a binary factor (no-noise = 0, noise = 1). The motivation and exhaustion variables both corresponded to the respective mean of the motivation/exhaustion values from the second and third assessment points (in the middle of and at the end of the story). The latent mind-wandering variable was generated via three parcels (Little et al., 2002). The first parcel constituted of a mind-wandering sum score for the assessment points 1, 4, 7, 10, 13, 16. The second parcel included assessment points 2, 5, 8, 11, 14, 17, and the third parcel points 3, 6, 9, 12, 15, 18. Finally, the comprehension variable also included three parcels which took into account the sum of correct answers to all 14 comprehension questions (first parcel: questions 1, 4, 7, 10; second parcel: 2, 5, 8, 11, 13; third parcel: 3, 6, 9, 12, 14). We chose to create three parcels per latent factor to allow for the independent estimation of factor loadings on the resulting latent mind-wandering and comprehension scores. Further, we allocated the items/assessment points to the parcels in a way that would consider the temporal trend of the assessed variables (see time-on-task effects in Table 3).

**Fig. 5:**
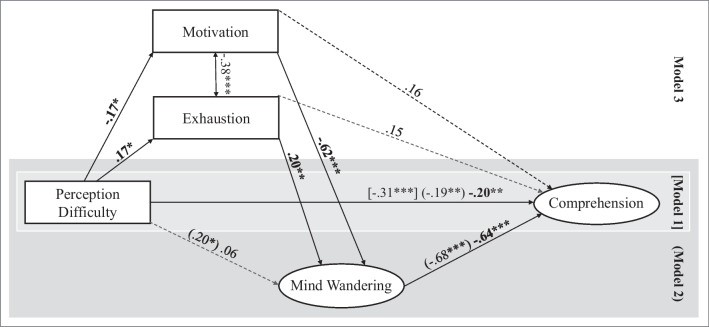
Structural path model of the relationship between perception difficulty and comprehension. *Note* The figure displays mind wandering as one possible mediator of the relationship between perception difficulty and comprehension as well as motivation and exhaustion as possible mediators of the relationship between perception difficulty and mind wandering. The mediators were added in a stepwise procedure from Model 1 to Model 3. Numbers represent standardized regression coefficients. Dashed lines indicate non-significant paths. The coefficient of Model 1 is displayed in brackets; the coefficients of Model 2 are displayed in parentheses. ***p < 0.001; **p < 0.01; *p < 0.05

In a first step (Model 1, Fig. 5), comprehension was regressed on perception difficulty. The observed negative effect of perception difficulty on comprehension in Model 1 got smaller but was still significant when adding mind wandering to the model in a second step (Model 2, Fig. 5). This indicated a partial mediation with a significant indirect effect of perception difficulty on comprehension via mind wandering (*b* =  − 0.13, *p* = 0.022). Finally, as possible mediators of the relationship between perception difficulty and mind wandering, motivation and exhaustion were included in the full model (Model 3, Fig. 5). The indirect effect via motivation (*b* = 0.11, *p* = 0.028) was significant, while that of exhaustion was not *(b* = 0.03, *p* = 0.073). The direct effect of perception difficulty on mind wandering that had been significant in Model 2 became non-significant in Model 3, indicating a full mediation. Interestingly, after adding mind wandering, motivation, and exhaustion to the model, perception difficulty still significantly related to reading comprehension.

## Discussion

The present study aimed to test whether an increase in perception difficulty results in task-focus benefits (i.e., less mind wandering and better text comprehension) or costs (i.e., more mind wandering, less motivation, and worse text comprehension). In line with the cost hypothesis, participants who were presented with a noisy version of a Sherlock Holmes novella reported more mind wandering and performed worse in a later comprehension test compared to those who read or listened to the novella without noise. We further established that motivational factors play a mediating role in the relationship between perception difficulty and mind wandering.

### Mind wandering

Even without increasing the perception difficulty of the material, reading and listening are complex higher-level cognitive tasks (Feng et al., 2013; Gernsbacher et al., 1990; Kintsch & van Dijk, 1978). Reducing readability or audibility increases the perception difficulty of the task and may lead to overload costs like text disengagement and susceptibility to distractors (e.g., mind wandering). In line with this idea, we found increased mind-wandering levels for the participants in both noise conditions independent of the presentation format.

#### Motivation as mediator

Failed situational-model building as well as increased cognitive demands could further lead the reader/listener to believe that focused reading/listening is not “worth the effort.” Such processes could eventually decrease motivation as has been previously demonstrated for situations with increased semantic processing difficulty (Kahmann et al., 2021; Soemer & Schiefele, 2019). Our study demonstrates that Gaussian noise can elicit similar motivational decrements. In our study, increasing perceptual processing difficulty decreased motivation and increased exhaustion during reading/listening. Motivation further mediated the positive relationship between perception difficulty and mind wandering. We assume that the relationship between perception difficulty and motivation might additionally be mediated by situational-model building failure and enhanced cognitive effort. However, this hypothesis should be tested in future studies before firm conclusions are drawn.

It is further possible that the sequence of cognitive and motivational processes differs from the one described in our path model, in which mind wandering was the consequence of low motivation. However, a failure to sustain attention on a task might also be the cause of decreasing motivation. When our minds drift, we might be more likely to make mistakes, become frustrated, and experience negative emotions such as boredom (e.g., Hunter & Eastwood, 2018) or apathy, leading to a decrease in motivation. Still, since there is also previous empirical evidence for increased motivation reducing mind-wandering rates (e.g., Seli et al., 2019), it is sensible to assume that the relationship between motivation and mind wandering is likely to be bidirectional. While this relationship is highly interesting not only for applied purposes (e.g., in an educational setting), it is not the main focus of this article (for a more in-depth exploration of the role of reading motivation, please see Soemer et al., 2023).

#### Resource competition versus overload

Other than hypothesized in our preregistration, we found no statistical evidence for our kind of perception-difficulty manipulation to result in task-focus and comprehension benefits. This benefit-assumption was based on the idea of a limited pool of attentional resources that are shared by task and distractor processing. We hypothesized that increasing a task’s perceptual demands might bind attention to the task itself thereby leaving less resources available for the processing of wandering thoughts (Faber et al., 2017; Forster & Lavie, 2009). However, this rationale does not seem to hold true when Gaussian noise (of a certain intensity) is added to reading or listening material. At first glance, this seems to be at odds with the results from Faber et al. (2017) who found a mind-wandering decrease when the participants read a disfluent, perceptually more demanding, text. However, the discrepancy in results might be explained by the kind or intensity of the perception-difficulty manipulation.^8^ It is possible that task-focus decrements due to increased perception difficulty emerge from problems with situational-model building, increased cognitive effort, and an accompanying motivational decline. For such mental processes to unfold, perception difficulties might have to be of a certain strength to be able to affect situational-model building and the perceived effort. Faber and colleagues reported that their difficulty manipulation was rather subtle and that participants did not subjectively perceive the reading task to be more difficult or effortful when the text was disfluent. Our manipulation check, however, revealed that participants in the noise conditions reported a higher perceived difficulty to follow the storyline than participants in the no-noise conditions. Thus, the manipulation of perception difficulty may have been considerably stronger in our work than in the study by Faber and colleagues.

For moderately increased perception-difficulty levels, task-focus benefits (i.e., decreased mind-wandering levels) may exist. At a certain point on the difficulty spectrum, however, a tipping point might be reached. If perception becomes overly difficult, situational-model building might be likely to suffer leading to a motivational decline and a higher distractibility. Similar ideas concerning U-shaped effects of task difficulty on mind wandering have been previously proposed for math-problem solving (Randall et al., 2014) and word-pair studying (Xu & Metcalfe, 2016). Further research investigating processing difficulty manipulations in a more fine-grained fashion is necessary to test for such a U-shape concerning perception difficulty in reading tasks. From an applied perspective, this could help find just the right perception-difficulty level for maintaining an on-task focus. If, for moderate difficulty levels, distractor processing is attenuated in a bottom-up and stimulus driven fashion (compare, Forster & Lavie, 2009; Lavie, 2005; Lavie et al., 2004), moderately increasing the perception difficulty would represent a highly feasible mind-wandering intervention method.

Whether increasing a task’s perception difficulty influences mind wandering in a certain direction might not just be dependent on the difficulty level but also the currently ongoing task. Reading or listening-comprehension tasks might be highly likely to evoke task-focus costs (especially with high levels of perception difficulty) because they are already complex and resource-demanding in themselves (Feng et al., 2013; Gernsbacher et al., 1990; Kintsch & van Dijk, 1978; Smallwood, 2011). Adding even more demands to such tasks could represent the proverbial final straw that results in a breakdown of the situational model. In turn, the beneficial features that this model can provide to comprehension later in the narrative (Smallwood, 2011) could be impacted. Relatively simple sustained-attention tasks (in mind-wandering research, often n-back or sustained-attention-to-response tasks) might, however, be less susceptible for task-focus costs due to increased (perception) difficulty. In fact, participants’ task focus even seems to benefit from high-difficulty levels. For such tasks, a decrease in mind-wandering rates is frequently reported in high-difficulty (often manipulated by increasing working-memory demands) conditions (e.g., Rummel & Boywitt, 2014; Smallwood & Schooler, 2006). To test for task-dependency effects of perception difficulty, future research should use similar noise manipulations across a variety of different tasks and task complexities.

Situational-model building failures as our proposed explanation for increased mind-wandering levels due to noise certainly underly more basic cognitive mechanisms. Soemer and Schiefele (2019), for example, argue that the increase in attentional failures due to enhanced semantic text difficulty might be a consequence of difficulties to maintain attention on the more difficult text versions. Such basic attentional or executive-control processes are inarguably involved in situational-model building, but also play a role during tasks other than reading. Investigating such processes in more detail might thus be a great opportunity to reconcile difficulty effects on mind wandering during various tasks.

#### Internal versus external distraction

In the present work, we conceptualized mind wandering as task-unrelated thoughts, without differentiating between internally (stimulus-independent, such as worries or memories) and externally (stimulus-dependent, such as thoughts triggered by a nearby conversation) generated content. Internal and external distractions can be seen as two separate cognitive phenomena that might, however, underly a general distractibility trait (e.g., Forster & Lavie, 2014, 2016). We did not directly test for distinct effects of perceptual difficulty on these two facets. Thus, we cannot rule out the possibility that our noise manipulation—even though it was monotonous and without meaningful content—might have attracted attention itself as a source of external distraction. Therefore, future research should focus on the distinction between internal and external distraction, for example, by including an external-distraction answering option in the experience-sampling procedure.

### Reading and listening comprehension

In previous studies, mind wandering has repeatedly been found to increase the risk of comprehension failure during reading (e.g., Feng et al., 2013; Steindorf & Rummel, 2020) with recent meta-analyses supporting the stability of this effect specifically for the reading domain (Bonifacci et al., 2022) and more broadly within an educational setting (Wong et al., 2022). As, in the current work, mind wandering increased with perception difficulty, it was thus not surprising that the participants in both noise conditions performed worse on the comprehension test than the participants in the no-noise conditions. Negative effects of our difficulty manipulation were therefore not only present on the mind-wandering, but also the comprehension variables. Within the path-model analyses, mind wandering was found to be a partial mediator of the relationship between perception difficulty and comprehension. The direct effect of perception difficulty on comprehension, however, remained statistically significant (even when adding the motivational variables in the full model). There is thus something unique about perception difficulty that explains comprehension variance above and beyond the other variables assessed in the present study. It is possible that some information from the story was not processed due to the Gaussian noise even when the participants were paying attention. The noise may have made several words almost unreadable or inaudible, so that some parts of the story could not be picked up and stored for later comprehension testing.

Within our linear regression models, we differentiated between fact-based and inference-based comprehension. Reading or listening to the novella with added noise resulted not only in less knowledge about story events, but also in worse inferences about the identity of the villain and the storyteller. To test effects of perception difficulty on fact-based comprehension, we had positioned the thought probes strategically so that they appeared right after an event that was referred to in one of twelve fact-based comprehension questions. Our results revealed that mind wandering at such an event increased the possibility that this specific event is not remembered at a later point in time.

Higher general mind-wandering levels while reading or listening also resulted in worse inference-based comprehension. When we added the mind-wandering variable to the inference-based regression model, the perception-difficulty predictor was no longer significant indicating that the difficulty effect was fully driven by mind wandering: Perception difficulty induced higher mind-wandering levels which hampered the integration of several story events that was necessary to identify the villain and the storyteller. This is in line with the results from Smallwood et al. (2008) and is consistent with the hypothesis that problems with situational-model building occurred under high perception-difficulty conditions. It is, however, a limitation that the inference-based comprehension measure only comprised two questions. Situational-model building and its relation to mind-wandering and comprehension processes under noise conditions should be further investigated. Tracking the time course of inference-based knowledge similar to our fact-based measure could allow for a closer investigation of when and why situational-model building is hampered or even fails.

### Presentation format

A further aim of our study was to understand whether the modality of presentation plays an important role in mind wandering and comprehension. As stated in the Introduction section, little research has previously been conducted investigating mind wandering during listening tasks (but see Konu et al., 2021), and none of the few studies investigated effects of perception difficulty. We conducted this study assuming that listening is of similar complexity as reading, and that similar processes must be undergone for successful reading and listening comprehension (Gernsbacher et al., 1990). Thus, we did not expect (large) differences between the conditions that differed in presentation format. However, reading and listening might be similar but not identical processes and, as Rogowsky et al., (2016, p. 1) stated, “there is a surprising lack of empirical research that directly evaluates the effect of mode of input on comprehension.” The present work thus contributes to the investigation and understanding of the (in)comparability of attention and comprehension processes between different modes of input.

In general, we found similar effects of noise on our main variables of interest (mind wandering and comprehension) for the visual and the auditory conditions. Concerning main effects of presentation mode, there was numerically more mind wandering in the auditory conditions, but our analyses did not reveal statistically significant differences between the presentation formats on this variable. Comprehension, however, was significantly affected by the presentation format with worse fact-based comprehension in the auditory conditions. This might imply that listening does indeed lead to worse comprehension than reading. One possible explanation might be that episodes of inattention (e.g., mind wandering) impact the listener more than the reader. A reader might catch her drifting thoughts just in time to re-read a certain sentence. A lister is not granted that opportunity: As soon as a sentence is spoken, it is gone. There is no possibility for re-listening.

However, it is also possible that there is no general reading (vs. listening) advantage and that the group differences in our study emerged due to the employed methods. The presentation duration in the reading conditions was fixed and adjusted to the listening times in the auditory conditions resulting in a possibly unnaturally slow reading experience. This might have given the visual participants a comprehension advantage by merely allowing them some extra time to re-read passages independently of their current state of thought. Ultimately, more research is needed to fully understand the role the modality plays for attention and comprehension processes in noise as well as no-noise environments. From an applied perspective, investigating modality effects becomes all the more important given the popularity of podcasts, audiobooks, and the like not only for leisure but also educational purposes.

### Applied implications

In the present work, perception difficulty was operationalized as Gaussian noise. In everyday life, we find many different factors that interfere with our ability to perceive and process visual and auditive information accurately: Illegible handwriting, printouts with poor contrast, poor lighting or bright sunlight, or a document scanned in low resolution are just a few examples of daily perceptual noise which make it difficult to read a given text. Similarly, processing of auditive information can be impaired, for example, due to poor audio quality (e.g., a bad connection during Zoom meetings), traffic or construction noise, or people talking in the background. Our results suggest that, when continuously present, daily-life perceptual difficulty can increase the occurrence of task-unrelated thought. In turn, this increase most definitely goes along with performance decrements. The mind-wandering literature is unanimous that high levels of task-unrelated thoughts hamper comprehension.

Our work suggests that high-intensity noise should be avoided in situations where text comprehension is key. In educational settings, retaining information from texts is a critical skill for academic success. Similarly, good text comprehension is required for success in many different jobs and professions (teachers, doctors, lawyers, journalists, etc.). Also, when reading for fun, when having conversations with others, when reading the instructions for putting together a piece of furniture, or when listening to one’s favorite comedy podcast, more frequent mind wandering due to perceptual noise can represent a source of disturbance and error. Since we tested only one kind, future research will have to determine which other kinds of perception difficulty impair task focus and comprehension in various situations.

## Conclusion

In our everyday life, there is often noise accompanying the information that we want to process. What happens to our task focus when a story is not easily perceptible? We examined the influence of one type of perception difficulty (Gaussian noise) on mind wandering during reading and listening and the comprehension of a short crime novella. Our arguably rather strong manipulation of perception difficulty increased mind-wandering levels and impaired comprehension. As the influence of perception difficulty on mind wandering has not been investigated extensively so far, the present study contributes new evidence to the yet-to-be-established big picture.

## Footnotes

1 Interestingly, for simple cognitive tasks (often an n-back or sustained-attention-to-response task), a decrease in mind-wandering rates is frequently reported in high-difficulty (versus low-difficulty) conditions (e.g., Rummel & Boywitt, 2014; Smallwood & Schooler, 2006). It is noteworthy that processing difficulty influences mind wandering in opposing directions when it is added to simple cognitive versus reading tasks.

2 We assume that once situational-model building has failed or is impaired, it is unlikely to be repaired (unless re-reading of passages is possible and actually performed). This impairment will thus be “dragged along” until the end of a reading phase (maybe even getting worse with time).

3 Participants were allowed to take part in the experiment on tablet computers, laptops and computers, but not on smartphones.

4 After the general mind-wandering question, we additionally asked participants whether their thoughts have been [1] detailed and specific, [2] in the form of images, [3] in the form of words, and [4] spontaneous (in contrast to deliberate). Participants answered these questions on a 4-point scale from “*not at all*” to “*completely*.” These questions were adapted from Sormaz et al. (2018) and are not of central interest to the current research questions. Participants’ means aggregated across all 18 assessment points on [1], [2], and [4] did not differ between the experimental conditions. For [3], there was a significant main effect of the presentation format, *F(*1,171) = 6.51, *p* = .012, η_p_^2^ = .04, and a significant interaction of format and difficulty level, *F(*1,171) = 5.59, *p* = .019, η_p_^2^ = .03. There was no statistical evidence for a group difference between auditory and visual presentation for the noise conditions, *t(*85) < 1.00, *p* = .897, *d* = 0.03, but for the no-noise conditions, *t(*86) = 3.39, *p* = .001, *d* = 0.72, with thoughts being more in the form of words in the visual (*M* = 2.44, *SD* = 0.66) than the auditive condition (*M* = 1.97, *SD* = 0.64).

5 All participants were asked to fill out German versions of the Cognitive Failures Questionnaire as a measure of general daily-life distractibility (Broadbent et al., 1982) and the Mind Wandering Questionnaire as a measure of internal daily-life distractibility (Mrazek et al., 2013). As these questionnaires are not of central interest to the current research question, they will not be mentioned further.

6 We followed the guidelines originally proposed by Jeffreys (1961) for the interpretation of Bayes factors: BF 1–3: anecdotal support; BF 3–10: substantial support; BF 10–30: strong support; BF 30–100: very strong support; and BF > 100: decisive support.

7 We tested the proportional odds assumption for this ordered regression model. The Brant–Wald test (χ^2^ = 1.05, *df* = 2, *p* = .59) suggested that the effect of perception-difficulty level on inference-based comprehension was the same when comparing it (i) for those who answered two questions correctly with those who answered one correctly and (ii) for those who answered one question correctly with those answered none correctly.

8 The present study and the study by Faber and colleagues (2017) further differed in the employed reading materials. Faber and colleagues used educational texts that were about three and a half times shorter than the crime novella that we used. Features of the reading material might influence cognitive and motivational variables and, in turn, mind-wandering reports.
